# Orthographic knowledge mediates the link between environmental print attention and early reading ability

**DOI:** 10.3389/fpsyg.2026.1852070

**Published:** 2026-07-23

**Authors:** Ying Xiao, Tianying Qing, Huidong Xue, Jing Zhao, Licheng Xue

**Affiliations:** 1 School of Preschool Education, Hangzhou Polytechnic, Hangzhou, Zhejiang, China; 2 Jing Hengyi School of Education, Hangzhou Normal University, Hangzhou, Zhejiang, China; 3 Zhejiang Philosophy and Social Science Laboratory for Research in Early Development and Childcare, Hangzhou Normal University, Hangzhou, Zhejiang, China

**Keywords:** attention, environmental print, eye-tracking, mediating effect, orthographic knowledge, preschool children

## Abstract

Children are exposed to a vast amount of environmental print (EP, such as “KFC” on billboards) at an early age. Attention to words in EP closely correlates with children’s reading ability. However, how children’s attention to words in EP is related to their early reading ability remains unclear. We addressed this issue by examining the mediating effect of orthographic knowledge on the association between children’s attention to words in EP and their reading ability. Results revealed that children’s reading ability was closely associated with both their attention to words in EP and orthographic knowledge. Furthermore, children’s attention to words in EP was significantly correlated with their orthographic knowledge. Importantly, orthographic knowledge was found to serve as a mediator in the association between attention to words in EP and reading ability. These findings advance the understanding of the potential role of EP in early reading development.

## Introduction

Preschool children are frequently exposed to environmental print (EP) containing visual words (e.g., “KFC” on advertising boards) in daily life. As a functional, ubiquitous, and appealing stimulus, EP increases opportunities for children to interact with words embedded in commercial logos and provides some of their earliest print experiences (Neumann et al., 2012; Whitehurst and Lonigan, 1998). Previous training studies have demonstrated that children’s engagement with words in EP facilitates the development of reading ability. For instance, prior training interventions have indicated that employing EP as a training tool effectively promotes the development of literacy skills in young children (Haney and Hill, 2004; Neumann, 2014; Roberts et al., 2005; Salewski, 1995). After completing an eight-week EP-based training program, preschool children in the experimental group exhibited significantly better performance on reading assessments than their peers in the control group (Wepner, 1985). Similarly, Neumann et al. (2013) implemented an eight-week training intervention, in which children were randomly assigned to three groups: an EP group, a standard print group (with identical words labels presented in black Century Gothic font), and a control group. Results indicated that following the training intervention, children in the EP group achieved higher scores on reading tests than those in the standard print group, whereas no significant difference in reading performance was observed between the standard print group and the control group (Neumann et al., 2013). A core premise underlying these training studies is that attention to words in EP is critical, given that attention to words serves as a key contributor to the development of children’s reading ability (Strandberg et al., 2022). Consistent with this theoretical framework, a recent study employing eye-tracking technology by Qing et al. (2022) revealed that children who allocated a greater percentage of fixation time to words in EP and exhibited shorter latency of the first fixation performed better on word recognition test. In other words, children who directed more attention to words in EP demonstrated higher reading ability. Furthermore, attention to words in EP constitutes a major factor predictor of individual differences in children’s reading ability (Qing et al., 2022). Collectively, these findings indicate that attention to words in EP is related to the development of reading ability in young children. This body of evidence also raises a fundamental question: how does attention to words in EP is related to reading ability in preschool children? Answering this question may further elucidate the role of EP in early reading development (Ehri, 2005; Ehri et al., 2006; Zhao et al., 2014).

According to theoretical models of early reading acquisition (e.g., Frith, 1986; Marsh et al., 1981; Mason, 1980; Ho et al., 2003a,b), orthographic knowledge may serve as a critical factor for explaining how attention to words in EP is related to reading ability in children. Frith’s (1985) three-stage model of early reading development posits that children achieve reading proficiency through three sequential phases: namely, the logographic, alphabetic, and orthographic stages. During the logographic stage, children rely on salient visual cues to identify words (e.g., using the golden “M” logo to recognize the word “McDonald’s” in EP). Fernández-López et al. (2021) provided evidence that preschool children already show sensitivity to letter position coding, indicating that a fundamental orthographic tuning mechanism is available and can be sharpened through repeated visual engagement with print. Such early-emerging sensitivity likely serves as the perceptual gateway through which sustained attention to words in EP translates into orthographic knowledge. Compared with alphabetic scripts, Chinese characters exhibit distinctive visuo-orthographic features. Leong et al. (2011) proposed that, in the Chinese writing system, orthographic knowledge refers to the understanding of the visual features of characters (such as strokes, radicals, and the rules governing radical positions) as well as the functions of components as semantic or phonetic radicals. Because Chinese word forms are hierarchically structured, orthographic awareness in Chinese encompasses several distinct levels. First, words are composed of strokes and radicals; consequently, learners must be able to recognize configurations containing strokes and radicals as words and to distinguish real words from non-existing ones. Second, because some radicals have flexible positions, learners must also acquire sensitivity to the legality of radical positions; different radicals can be combined into words with various structural types (e.g., top–bottom, left–right, enclosed), thereby requiring structural awareness. Orthographic knowledge develops hierarchically and progressively more precise (Castles et al., 2007; Levy et al., 2006). For Chinese preschool children, higher-level orthographic knowledge—such as sensitivity to radical positions and radical combinatorial rules—has not yet been acquired (Zhao et al., 2014). However, lower-level orthographic knowledge is already developing: preschoolers show a good grasp of stroke-level features and can distinguish stimuli containing stroke patterns (e.g., real words and stroke combinations) from stimuli lacking stroke features (e.g., line drawings) (Zhao et al., 2014). Early on, preschool children can perceptually differentiate real characters from word-like symbols, and this ability is significantly correlated with their reading ability (Ho et al., 2003a,b). As children gain increased perceptual experience with visual words, such as consistent exposure to words in EP, they gradually discern more internal features of visual word forms. For instance, children begin to recognize that words are composed of letters (strokes in Chinese words) rather than arbitrary visual symbols (line drawings). Accordingly, children develop orthographic knowledge and exhibit an improved ability to discriminate real words from symbol strings or even other visually similar words (Pelli et al., 2006; Zhao et al., 2014). This ability constitutes a foundational skill for reading development (Ehri, 1995). Therefore, as children direct greater attention to words in EP, they accumulate more visual word exposure and gradually grasp the internal features of visual word forms. The acquisition of orthographic knowledge in turn further facilitates reading development in children (Conrad et al., 2013; Querido et al., 2021; Wang et al., 2014; Zarić et al., 2021). On the basis of this theoretical rationale, we hypothesized that orthographic knowledge served as a mediator in the association between children’s attention to words in EP and their reading ability.

Our hypothesis regarding the mediating effect of orthographic knowledge has received indirect support from existing literature. Previous research has demonstrated that extensive perceptual experience with words in EP facilitates the early acquisition of orthographic knowledge (Fletcher-Flinn and Thompson, 2004; Zhao et al., 2014, 2018). Orthographic knowledge refers to the understanding of orthographic rules within a language, such as the regularities and conventions governing letter combinations (Conrad et al., 2013). This construct has most commonly been assessed using the word-likeliness task developed by Siegel et al. (1995), in which participants are required to judge whether a given stimulus is the real word. In other words, the orthographic knowledge is typically reflected in the ability to distinguish real words from other word-like stimuli in a lexical decision task, where participants are instructed to decide whether one stimulus is a real word or not (Apel, 2011; Conrad et al., 2013). Orthographic knowledge is generally acquired through children’s consistent exposure to words (Barker et al., 1992; Stanovich and West, 1989). Accordingly, extensive visual engagement with EP supports the development of orthographic knowledge in preschool children (Zhao et al., 2014; Zhao et al., 2018; Qing et al., 2022; Barker et al., 1992; Fletcher-Flinn and Thompson, 2004). In line with this theoretical account, prior empirical studies have reported significant correlations between children’s experience with EP and their orthographic knowledge (Lomax and McGee, 1987; Stanovich and Cunningham, 1992). For instance, Lomax and Mcgee (1987) measured orthographic knowledge by asking children to distinguish letters from letter-like symbols, and assessed their experience with EP in an EP recognition task. The results revealed that children with greater EP experience showed higher orthographic knowledge (Lomax and McGee, 1987).

Furthermore, previous studies have documented positive correlations between orthographic knowledge and children’s reading ability (Conrad et al., 2013). Ho et al. (2003a,b) identified orthographic knowledge as a key component of Chinese word reading development. Previous studies has also demonstrated that orthographic knowledge constitutes a major predictor of individual difference in children’s reading ability (Conrad et al., 2013; Querido et al., 2021; Wang et al., 2014; Zarić et al., 2021). In a recent longitudinal study, Zarić et al. (2021) found that children’s orthographic knowledge during the preschool stage significantly predicted their subsequent reading ability after school entry. Collectively, these findings imply the important role of orthographic knowledge in facilitating children’s reading development.

**Figure 1:**
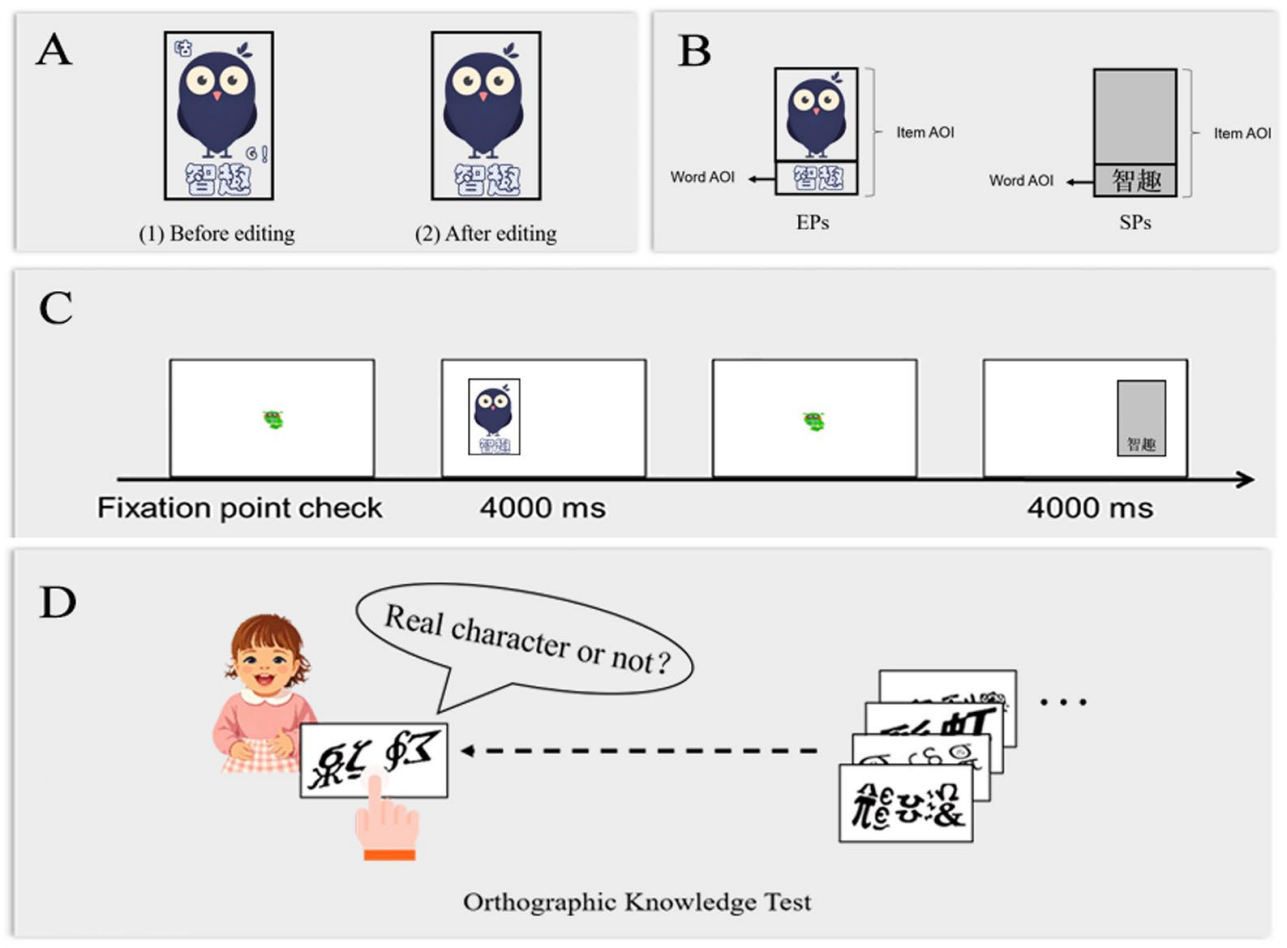
**(A)** An example of the actual environmental print in children’s daily life (1), and the environmental print used in this study after editing with Photoshop (2). **(B)** Samples of item- and word-AOI of environmental print. **(C)** A schematic diagram of the free viewing task. **(D)** An example of the orthographic knowledge test.

To further examine the hypothesis regarding the mediating effect of orthographic knowledge, we employed eye-tracking technique to measure children’s attention to words in EP (Neumann et al., 2015; Qing et al., 2022; Zhao et al., 2018). To explore specificities associated with words in EP (Puškarević et al., 2016), we also used the standard print (SP) condition as a control. In this control condition, all logo, color and font cues were removed, and the words were presented in a standard print font (see Figure 1B for an example). Children’s orthographic knowledge was assessed using a lexical decision task (Araújo et al., 2014; Hudson and Bergman, 1985). Additionally, we measured reading ability by using the word recognition test designed for preschoolers, which was widely used in previous studies (Chow et al., 2008). Finally, mediation analyses were conducted on the collected data to examine the relationships among children’s attention to words in EP, orthographic knowledge, and reading ability.

Based on results in previous studies (Qing et al., 2022; Zhao et al., 2018), we expected that attention to words in EP would be closely correlated with children’s ability to read words. According to previous findings (Conrad et al., 2013; Zarić et al., 2021), we also expected that orthographic knowledge would correlate with reading ability, while attention towards words would be correlated with orthographic knowledge (Ho et al., 2003a,b). Most importantly, we expected that orthographic knowledge would serve as the mediator between children’s attention to words in EP and their reading ability.

## Methods

The research protocol reported here was approved by the human research reviewing committee of the Hangzhou Normal University. We obtained oral informed consent from children and written informed consent from parents or other legal guardians.

### Participants

By posting recruitment information in a local preschool, 58 parents agreed to let their children participate in this study. The 58 participants in this study were Mandarin-speaking children. One participant’s data was excluded as he dropped out in the middle of the study. Two participants’ data were excluded because their head movements affected the quality of eye movement data. The remaining valid participants were 55 (24 girls, 31 boys; 22 in the second year of preschool, 33 in the third year of preschool) with a mean age of 69.6 months (range 62–75 months; *SD* = 5.9) and had normal vision. Preschool children in mainland China do not receive any conventional reading training as in elementary school. They mainly have contact with words in their daily life such as words in environmental print and picture books.

### Stimuli

Environmental print was selected according to a five-point rating scale of familiarity (1 = never to 5 = frequently) in which 24 logos were evaluated by 254 parents and teachers. Parents were asked to rate their children’s familiarity with EP. The EP with the top 10 familiarity was selected, which were the logos of Oreo, Cai Hong, Gong Gong Ce Suo, Hai Di Xiao Zong Dui, Jin Zhi Xi Yan, Ken De Ji, Wang Wang, Wang Zai Niu Nai, Xiao Zhu Pei Qi, and Xiong Chu Mo (see Supplementary Table S2). The score of familiarity evaluation was between 4.475 and 3.839. To ensure that there was only one word area of interest in the image, we used the Photoshop to remove any form of words except the prominent words in each image (see Figure 1A for an example). To clarify the specific role of EP in children’s early reading development, two versions were used in the present study (see Figure 1B for an example): (a) environmental print (EP): words were presented in a full context including logo, color and font cues; (b) standard print (SP): removing the logo, color and font cues of EP, keeping the words in EP but presenting those in a standard print font (Song). The written brand names within the target AOIs were matched in font size (i.e., the word-AOI sizes were identical) and visual complexity (i.e., stroke count and structural complexity were equivalent). The range of the images’ visual angle was from 19.78° to 34.10°, and the viewing distance was 65 cm.

### Apparatus

A 19-inch color LED monitor was used to present all the stimuli. Experiment Builder software (SR Research, Ottawa, ON, Canada) was used to build the experimental script. The visual attention on the visual display was recorded with an eye-tracker (EyeLink 1,000, SR Research, Ottawa, ON, Canada). The monocular sampling rate was set to 500 Hz, and the tracking accuracy of the eye-tracker was reported to be 0.25^°^ or better.

### Procedure

Free viewing task. The experiment was carried out in a single quiet classroom in the preschool. To measure children’s visual attention towards words in EP and SP, a free viewing task was used in the eye-movement experiment (see Figure 1C, also see Neumann et al., 2015; Qing et al., 2022; Zhao et al., 2018 for more discussion). Twenty images, including ten EP and ten SP, were presented against a white background. The EP and SP were rendered once to the left and right of the same position from the center of the screen, so we presented the images twice throughout the experiment. The viewing distance was about 65 cm maintained by a rest. A nine-point calibration grid was used. Participants were instructed to fixate on a cartoon frog in graphic interchange format on a white background.

All images were presented randomly. Moreover, the location of each presentation was not fixed; the image could appear either to the left or to the right of the central fixation point. Camera calibration was checked after each image was displayed for 4,000 ms. Children had no idea about the main purpose of the study. They were asked to freely view the images on the monitor, with the instruction “Some images will present on the monitor, and what you need to do is to freely view these images whenever they appear.”

### Assessments

Word recognition test. A word recognition test developed for preschoolers (Chow et al., 2008) was used to test children’s reading ability. Specifically, the test with 61 items requires children to read them aloud one by one in order. The difficulty of these items was arranged in increasing difficulty. The maximum score is 61 and the minimum is 0. If the participant incorrectly answered 10 items in a row, the test was stopped immediately.

Orthographic knowledge test. Similar to the procedure used in previous studies (Liu et al., 2017; Taft and Zhu, 1997; Tong et al., 2009; Zhao et al., 2019), a lexical decision task was used to assess children’s orthographic knowledge, in which they were asked to identify whether an item was real Chinese word or not. As noted earlier, Chinese preschool children have not yet acquired higher-level orthographic knowledge such as sensitivity to radical positions and radical combinatorial rules (Zhao et al., 2014). However, lower-level orthographic knowledge, such as knowledge of stroke-level features, is already developing. Therefore, the present study focused on assessing low-level orthographic feature knowledge. Two types of stimuli were used, that were real words and symbol combinations. Specifically, 10 real words were the word in the EP with the removal of the color and logo cues (e.g., 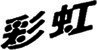), and 10 symbol combinations constructed with meaningless symbols (e.g., 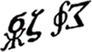). The symbol combinations were well matched with real words in visual complexity, structure, and number of lines. All stimuli were presented in black color on a white background card, and the order was completely randomized. Children were asked to decide whether stimuli were real Chinese *word* or not (see Figure 1D). The score was the number of correct responses (i.e., “yes” response to real words and “no” response to symbol combinations). The maximum score of each type is 10, and the minimum score is 0. Prior to the formal experiment, we conducted a pilot study and confirmed that the children were able to understand the instructions and task requirements. The stimuli in the lexical decision task overlapped with those in the eye-tracking task. The lexical decision task was administered approximately one month after the eye-tracking task. The interval may have reduced immediate repetition or carryover effects. In addition, the lexical decision task required children to make a judgment on a single picture and respond verbally, the test duration was short, and it was administered approximately one month after the eye-tracking task. Therefore, it was minimally influenced by working memory, response demands, or general attentional control.

The three assessments were conducted in a quiet classroom in the kindergarten. The eye-movement experiment took approximately 5 min. To minimize the influence of memory, we measured orthographic knowledge and reading ability individually after all participants had completed the eye tracking task. All children completed the tasks in the same order. In the end, each child received a toy as a present for his or her participation.

### Data processing

Eye movement data were extracted using Eyelink Data Viewer (SR Research, Ottawa, ON, Canada). Fixations associated with blinks were cleaned, fixations near to each other were combined (amplitude threshold = 1.5^°^), and those with fixation durations shorter than 50 ms were excluded from the data (Qing et al., 2022; Zhao et al., 2018).

To analyze children’s attention toward words in the EP, we defined the area of interest (AOI) for each EP and SP image (see Figure 1B). As shown in Figure 1B, the word AOI was drawn closely surrounding the visual words in the image, and the item AOI contained the entire image. We used rectangular AOIs drawn tightly along the edges of the text; this procedure was applied consistently across all stimuli, and the AOI sizes were identical for EP and SP. The analysis of eye movement data was based on the fixations within the word and item AOI, the fixations outside the item AOI were excluded.

Two independent indexes were considered in the eye movement data analysis as different indexes reflect different aspects of attention (Qing et al., 2022; Zhao et al., 2018): (a) The latency of the first fixation, the time from stimulus presentation to the participant’s first fixation on the word AOIs, which reflects the speed at which the participants pay attention to words (Hoffman and Subramaniam, 1995; Vervoort et al., 2013); (b) The percentage of fixation durations, the percentage of the duration of fixations on the word AOIs over all the duration of fixations on the monitor, which reflects attentional maintenance to the stimulus (Vervoort et al., 2013). To control the between-subject variations in total fixation durations, the ratios of eye movement data were considered (Neumann et al., 2015; Qing et al., 2022; Zhao et al., 2018).

According to the signal detection theory (Macmillan and Creelman, 2005), the sensitivity (*d’*) of the lexical decision task is analyzed and used to examine children’s orthographic knowledge. Compared with separate hit and false-alarm rates, d′ provides a composite index that comprehensively reflects children’s ability to discriminate real Chinese words from word-like symbols; therefore, we used d′ as the primary measure of orthographic knowledge. Sensitivity (*d’*) is calculated as the z-score of hits minus the z-score of false alarms. The z-score of hits and false alarms are the corresponding *Z* scores on the normal distribution curve for the hit rate and the false alarm rate. Specifically, the hit rate was defined as the percentage of “yes” response with real words (P (hit) = the percentage of “yes” response with real words / 10), and the false alarm rate was defined as the percentage of “yes” response with symbolic combinations (P (false alarm) = the percentage of “yes” response with symbolic combinations / 10). The range of sensitivity (*d’*) is −4.65 to 4.65.

## Results

### Descriptive statistics

Table 1 shows the descriptive statistics of children’s attention toward word AOIs of EP and SP under the two indexes, orthographic knowledge and word reading score. The results of the distribution examination showed that the absolute skewness or kurtosis of all variables was below 1.96, except for the percentage of fixation durations on word AOIs of EP and the latency of the first fixation on word AOIs of SP. The variable was logarithmically transformed to enhance pairwise linearity and to decrease extreme skewness and kurtosis (Tabachnick and Fidell, 2007), and was normally distributed after logarithmically transformed. Accordingly, in subsequent analyses, we applied a log10 transformation to the percentage of fixation duration on word AOIs of EP and the latency of the first fixation on word AOIs of SP; all descriptive statistics and results (including those presented in tables and figures) for these two variables are based on the transformed data. The latency of the first fixation on word AOIs of EP, the percentage of fixation duration on word AOIs of SP, word reading scores, and orthographic knowledge (d′) were not transformed; all statistics and results for these variables are based on raw data.

**Table 1:** Means, standard deviations, and correlations among measures.

Measures	Skewness	Kurtosis	*M* (*SD*)	1	2	3	4	5	6
1. Latency of the first fixation on word of EP	0.373	0.122	1241.963 (359.090)	1					
2. Latency of the first fixation on word of SP	1.292	−0.658	2.792(0.172)	–	1				
3. Percentage of fixation durations on word of EP	−0.028	0.576	−0.767 (0.207)	–	–	1			
4. Percentage of fixation durations on word of SP	0.475	−1.334	0.417 (0.138)	–	–	–	1		
5. Orthographic knowledge (d*’*)	−0.721	−1.803	2.030 (1.426)	−0.440^**^	*−0.038*	0.303^*^	*0.247*	1	
6. Word reading score	1.860	−1.292	21.182 (18.450)	−0.556^**^	*−0.043*	0.370^**^	0.346^**^	0.589^**^	1

Italicized values indicate partial correlation coefficients controlling for age. ^*^p < 0.05, ^**^p < 0.01.

Although formal literacy instruction is not provided in mainland Chinese kindergartens, variability in the home literacy environment leads to substantial individual differences in preschool children’s character recognition abilities. The word reading scores in the present study confirm that the sample included both prereaders and emerging readers. The distribution of word reading scores approximated normality. To examine mediation pathways at the individual level, we conducted correlational and mediation analyses treating word reading as a continuous variable rather than creating discrete groups.

The lexical decision task had a difficulty index of 0.437, indicating a moderate level of difficulty. The *d*′ scores for orthographic knowledge were normally distributed, with no evidence of floor or ceiling effects. To further examine the task’s discrimination, we used an extreme-groups approach and compared d′ between the highest 27% and the lowest 27% of scorers. An independent-samples t-test revealed a significant difference (*t* = −24.462, *p* < 0.01), confirming that the task had good discriminative power.

### Correlation analyses

Table 1 shows Pearson’s correlation coefficients between children’s attention toward word AOIs of EP and SP under the two indexes, orthographic knowledge and word reading score. The sample included children aged 62 to 75 months, a developmentally meaningful range for early literacy, orthographic knowledge, visual attention, and task performance. In the correlational analysis, we included age in months as a covariate to more clearly delineate the relationships among orthographic knowledge, attention to words in EP, and reading ability. Results showed that children with shorter latencies before first fixation on a word in EP were better at distinguishing words from visual symbols and better at reading words. Children who fixated longer on words in EP had better orthographic knowledge and reading skills. Most measurements of attention towards words were associated with children’s orthographic knowledge (all *p*-values < 0.05), except for the latency of the first fixation to word AOI in SP (*r* = −0.038, *p* = 0.786) and the percentage of fixation durations on word AOIs of SP (*r* = 0.247, *p* = 0.072). In addition, most measurements of attention towards words were related to children’s reading ability (all *p*-values < 0.05), except for the latency of the first fixation to word AOI in SP (*r =* −0.043*, p =* 0.759). That is, the mediation effect of orthographic knowledge between attention to words in SP and reading ability was not statistically significant. As such, in the mediation analyses, we only examined the mediating role of orthographic knowledge in the relationship between attention to words in EP and reading ability under the two EP eye-tracking indices.

### Mediation analyses

Based on the results of the correlation analysis (see Table 1), we further examined our hypothesis that there would be a mediating effect of orthographic knowledge between children’s attention towards word AOI in EP and their reading ability.

To examine the aforementioned hypothesis, we used the program of Bootstrap. The bootstrap sample was set to 5,000 according to the program of mediation effect analysis (Zhao et al., 2010) and the bootstrap method for the mediating effect test (Preacher and Hayes, 2004). The indirect mediation effect is significant when 0 is not included in the 95 % confidence interval. The present study used the PROCESS macro (Hayes, 2013) of SPS 20.0 to assess the mediating effect. Similarly, we also included age in months as a covariate in the mediation analysis to more clearly examine the mediating role of orthographic knowledge between EP print attention and early reading ability.

**Figure 2:**
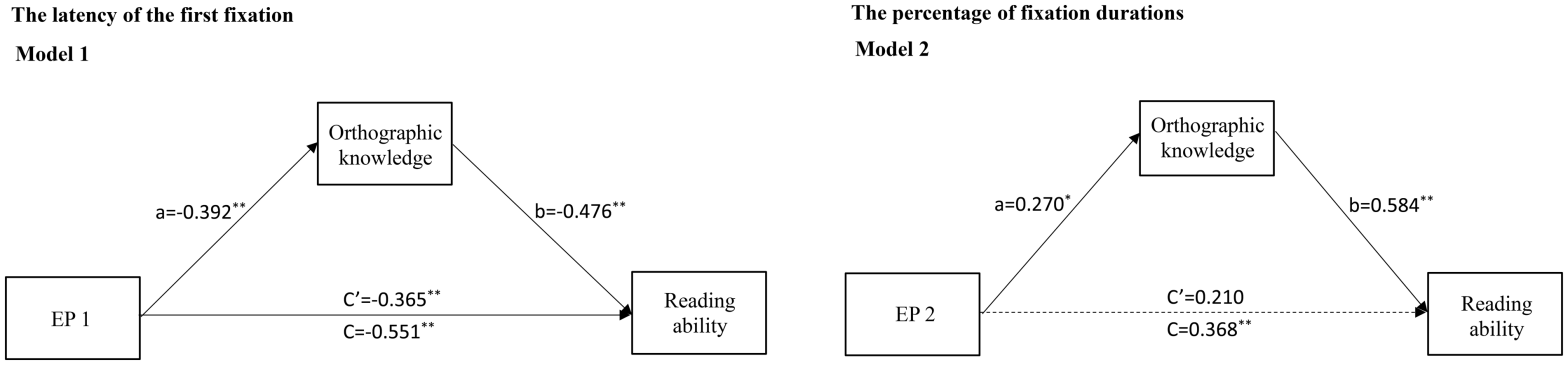
The mediating effect of orthographic knowledge between attention towards words in EP and reading ability in two indexes. *p < 0.05, **p < 0.01.

We first examined the effect of children’s attention towards words in EP reflected in the latency of the first fixation on reading ability. The regression model showed that children’s attention towards words in EP was a significant predictor of reading ability in this case (*β* = −0.551, SE = 0.006, *p* < 0.01). Second, we examined whether orthographic knowledge was a mediator linking children’s attention towards words in EP and reading ability. As shown in Model 1 in Figure 2, in predicting reading ability, orthographic knowledge and children’s attention towards words in EP were significant predictors (*β* = 0.476, SE = 1.657, *p* < 0.01; *β* = −0.365, SE = 0.006, *p* < 0.01, respectively). In predicting orthographic knowledge, children’s attention towards words in EP was a significant predictor (*β* = −0.392, SE = 0.0004, *p* < 0.01). As shown in Table 2, the indirect effect of orthographic knowledge in Model 1 was statistically significant.

**Table 2:** The direct and indirect paths through which children’s attention towards words in EP is associated with reading ability.

Direct and indirect paths	*β*	95% CI	
		Low	High
The latency of the first fixation			
Mediation model 1			
Path 1: EP 1 → Reading ability	–0.365^**^	–0.031	–0.007
Path 2: EP 1 → Orthographic knowledge → Reading ability	–0.186	–0.327	–0.062
The percentage of fixation durations			
Mediation model 2			
Path 1: EP 2 → Reading ability	0.210	−1.170	39.232
Path 2: EP 2 → Orthographic knowledge → Reading ability	0.158^*^	0.007	0.307

EP 1, the latency of the first fixation on word AOIs of EP; SP 1, the latency of the first fixation on word AOIs of SP; EP 2, the percentage of fixation durations on word AOIs of EP; SP 2, the percentage of fixation durations on word AOIs of SP. ^*^p < 0.05, ^**^p < 0.01.

Similarly, we tested the effect of children’s attention towards words in EP reflected in the percentage of fixation durations on reading ability. The regression model showed that attention was a significant predictor of reading ability in this case (*β* = 0.368, SE = 11.424, *p* < 0.01). As shown in Model 2 in Figure 2, in predicting reading ability, orthographic knowledge was a significant predictor (*β* = 0.584, SE = 1.656, *p* < 0.01) and children’s attention towards words was not a significant predictor (*β* = 0.210, SE = 10.197, *p* = 0.072). In predicting orthographic knowledge, children’s attention towards words was a significant predictor (*β* = 0.270, SE = 0.814, *p* = 0.026). As shown in Table 2, the indirect effect of orthographic knowledge in Model 2 was statistically significant.

To fully demonstrate the order of predictor (attention toward words in EP), mediator (orthographic knowledge) and outcome variables (reading ability), we further investigated the opposite relation: orthographic knowledge may mediate the relation between reading ability (predictor) and attention toward words in EP (outcome).

In predicting children’s attention towards words in EP, reading ability was a significant predictor (*β* = −0.458, SE = 2.787, *p* < 0.01) but orthographic knowledge was not a significant predictor (*β* = −0.193, SE = 40.145, *p* = 0.230). Further results show that the indirect effect of orthographic knowledge between reading ability and children’s attention towards words in EP was not statistically significant (95% CI = [−0.289, 0.101]). We also tested the effects of reading ability on children’s attention towards words in EP reflected in the percentage of fixation durations. The results showed that the indirect effect of orthographic knowledge between reading ability and children’s attention towards words in EP was not statistically significant (95% CI = [−0.175, 0.284]). The results showed that orthographic knowledge does not mediate the relation between reading ability (predictor) and attention toward words in EP (outcome).

To further examine to what extent the reported effects reflect children’s attention to the written brand names themselves, rather than differences in the visual characteristics of the stimuli, we re-ran the mediation models with stricter covariate controls (see the Supplementary material for detailed results). Reassuringly, the core pathways in the EP condition remained highly robust (demonstrating a strict full mediation effect), whereas the indirect pathways in the decontextualized SP condition failed to reach significance.

## Discussion

The present study investigated how attention towards words in EP is related to children’s reading ability. We found that children’s attention to words in EP was correlated with their reading ability and orthographic knowledge, while orthographic knowledge was associated with reading ability. Most importantly, we found the mediating effect of the orthographic knowledge on the association between attention to words in EP and reading ability.

Consistent with results in previous studies, we found that preschool children’s reading ability was associated with their orthographic knowledge (Liu and Liu, 2020; McBride-Chang and Ho, 2005; Shu et al., 2008; Yang et al., 2019), and attention towards words in EP (Blankenship et al., 2019; McClelland et al., 2013; Plaza and Cohen, 2007; Qing et al., 2022; Valdois et al., 2019). Furthermore, the results indicated that children’s attention to words in EP was positively associated with their orthographic knowledge. Previous studies have found children’s recognition of words in EP is correlated with their orthographic knowledge (Lomax and McGee, 1987; Stanovich and Cunningham, 1992). By using the eye-tracking technique, our findings extend this line of research and provide direct evidence that attention to words in EP is positively related to orthographic knowledge. Notably, as specified in the method section, the EP stimuli used in the free-viewing task overlapped slightly with those adopted in the orthographic knowledge test. To minimize the potential confounding effect of immediate repetition or carryover effect for the EP materials on their performance in the orthographic knowledge assessment, the orthographic knowledge test was administered individually to all participants approximately one month after they completed the free-viewing task. However, it is also critical to note that it cannot fully exclude episodic memory or prior familiarity with the same written forms.

The most important finding of the present study is that orthographic knowledge acts as a mediator between children’s attention to words in EP and their reading ability. Specifically, orthographic knowledge fully mediated the association between children’s attention to words in EP and reading ability when indexed by the percentage of fixation durations, whereas it exerted a partial mediating effect for the first fixation latency measure. One plausible account for this discrepancy is that the two eye-tracking indices reflect distinct facets of attention. As noted earlier, first fixation latency indexes the speed of attentional orientation, reflecting the initial viewing pattern upon stimulus onset (Gamble and Rapee, 2009). By contrast, fixation duration characterizes the viewing pattern following the initial orienting phase, reflecting attentional engagement and sustained processing, which corresponds to the late stage of attention allocation (Calvo and Lang, 2004). In-depth information processing relies on sustained attentional engagement, while rapid attentional orientation does not guarantee prolonged attention maintenance. Accordingly, orthographic knowledge exhibited a full mediating effect for the fixation duration index but only a partial mediating role for the first fixation latency index. It is also important to note that while our findings align with the letter-position sensitivity frameworks observed in alphabetic scripts (Castles et al., 2018; Fernández-López et al., 2021), this proposed perceptual pathway remains a theoretically plausible hypothesis for Chinese orthographic development. Given that we did not directly assess higher-level orthographic precision in the present study, future research is warranted to empirically validate how visual attention to environmental print translates into fine-grained orthographic knowledge in logographic writing systems.

Interestingly, orthographic knowledge yielded no significant mediating effect between attention to words in standard print (SP) and reading ability for the first fixation latency or the percentage of fixation durations indexes. This finding demonstrates the unique role of environmental print in early reading development. Unlike standard print, EP features vivid images, bright colors, and distinctive fonts that initially capture children’s visual attention. The ability to redirect focus toward the textual elements of EP without being distracted by salient non-print cues is beneficial for reading development. Notably, the present study further clarifies the question that how attention to words in EP is related to reading ability. Compared with SP, the defining feature of EP is its rich non-verbal visual context. Children’s rapid attentional allocation to words within EP not only directly boosts reading ability but also indirectly facilitates reading development by enhancing orthographic knowledge.

These findings advance the understanding of how attention to words in EP is related to children’s reading development. Environmental print provides children with abundant perceptual experience with visual word forms (Neumann et al., 2014; Neumann et al., 2015; Vukelich et al., 2019). Sustained attention to words embedded in EP facilitates children’s learning of the inherent regularities of written language, thereby is related to the development of orthographic knowledge. In the Chinese writing system, visual input is only recognized as character-conforming when strokes and radicals are structured in line with orthographic rules, and mastering such orthographic knowledge is central to learning to read Chinese word (Ho et al., 2003a,b). Children’s attention to words in EP thus is related to their understanding of orthographic knowledge. Our results were also consistent with Frith’s model of early reading acquisition (Frith, 1985), which posits that engagement with environmental print fosters orthographic development and subsequent reading proficiency. Collectively, these findings highlight the mediating role of orthographic knowledge in the link between attention to words in EP and children’s word reading ability, corroborating prior relevant research (Lomax and McGee, 1987).

Notably, the present findings were derived from Chinese-speaking children. Compared with logographic Chinese word, alphabetic words feature more transparent grapheme-phoneme correspondences, with visual word forms mapping more regularly onto phonological representations (Caravolas, 2004; Caravolas et al., 2001; Ouellette and Sénéchal, 2008; Vaessen and Blomert, 2013). Chinese literacy acquisition depends heavily on visual-orthographic processing, so exposure to EP may be particularly conducive to orthographic knowledge development for Chinese-speaking children. In contrast, children learning alphabetic scripts may prioritize phonological knowledge acquisition when engaging with environmental print. Although orthographic knowledge is also critical for alphabetic reading acquisition, other factors—especially phonological knowledge—may serve as key mediators (Perfetti et al., 2005; Qing et al., 2022; Yeh and Li, 2002). Therefore, a valuable avenue for future research is to examine whether orthographic knowledge similarly mediates the link between EP attention and reading ability among children learning alphabetic scripts. To deepen understanding of the role of EP in reading development, cross-linguistic studies targeting alphabetic readers are warranted.

The present findings carry preliminary practical implications for the use of EP in early literacy education; however, given that the current study did not employ longitudinal or training designs, these implications should be interpreted with caution and warrant future investigation. Previous studies have verified that EP promoted early reading development in both classroom teaching (Giacovazzi et al., 2021; Neumann, 2014; Neumann et al., 2013; Salewski, 1995; Wepner, 1985) and home literacy environments (Neumann, 2018; Neumann et al., 2009). For instance, Salewski et al. (1995) implemented a four-week EP-based training program for 3- to 6-year-old children, revealing that the experimental group outperformed the control group on both EP recognition and word reading assessments. In another study, Neumann and colleagues (Neumann, 2018) trained parents of 3.5-year-old children to conduct 30 min of weekly literacy interactions using EP over an eight-week period, and results showed that children in the experimental group achieved higher emergent literacy scores than the control group. These findings confirm that EP serves as an effective instructional tool to boost children’s reading ability and that adult guidance enhances children’s EP recognition skills. However, prior training studies have not specified the optimal focus of parent–child or teacher-child interactions involving EP. The present findings, which establish the mediating role of orthographic knowledge, suggest that parents and early childhood educators should leverage EP to target orthographic development. Specifically, adults can guide children to focus on the textual components of EP and help them distinguish real words from word-like non-words within environmental print, laying a solid foundation for formal reading learning. Practical activities include asking children to circle printed words within EP stimuli, or comparing standard EP with modified versions where words are replaced with meaningless symbols to identify differences and judge authentic environmental print. Such activities may foster orthographic knowledge acquisition, supporting children’s subsequent reading development.

Several limitations of the present study warrant mention. First, EP stimuli were selected based on parent/teacher familiarity ratings, but subjective familiarity alone may not fully control for factors that guide children’s attention. These logos may differ in exposure frequency, emotional valence, motivational relevance, attractiveness, color, visual salience, child-specific preferences, and personal experiences (Horner, 2005; Salgado-Montejo et al., 2014; Ahmad, 2021). Future research should systematically assess and control for these variables and, where possible, balance EP categories and other potential confound to ensure stimulus standardization. Although we attempted to minimize physical differences between EP and SP, and the core pathways in the EP condition remained highly robust which demonstrated a strict full mediation effect with stricter covariate controls, suggesting the reported effects reflect children’s attention to the written brand names themselves, rather than differences in the visual characteristics of the stimuli. We acknowledge that they inevitably differ in logo context, color, font, visual salience, and other properties, which are difficult to match completely. Second, the lexical decision task and free-viewing task shared overlapping stimuli. This creates direct overlap between the predictor-related stimuli and the mediator measure. Although the one-month interval between the tasks was intended to reduce immediate repetition or carryover effects, it is critical to recognize that residual episodic memory and pre-existing familiarity with the shared written items cannot be entirely ruled out. Therefore, our findings should be interpreted with caution regarding the extent to which they pure-play reflect independent orthographic knowledge. Future research should employ independent stimulus sets across tasks. Third, the present study only examined the mediating role of orthographic knowledge. The results indicated that orthographic knowledge mediated 38 to 69% of the variance in word reading ability, suggesting that attention to words in EP may also be related to other reading-related cognitive skills. To fully unpack the mechanism underlying EP-facilitated reading development, subsequent studies should incorporate additional potential mediating variables. Fourth, despite employing mediation analysis, the present cross-sectional design cannot establish definitive causal links among attention to words in EP, orthographic knowledge, and reading ability. Future longitudinal training studies are needed to provide direct causal evidence for the mediating effect of orthographic knowledge in the relationship between EP word attention and reading ability. Fifth, due to practical constraints, the present study was unable to collect quantitative data on home literacy environment, parental education, general language ability, and individual experience with environmental print. To gain a clearer understanding of the relationships among early reading development, orthographic knowledge, and attention to environmental print, future studies should measure these variables and incorporate them into correlational and mediation models. Finally, although we selected two representative eye-movement indices based on previous environmental print research (Neumann et al., 2014; Neumann et al., 2015; Zhao et al., 2018; Qing et al., 2022; Xue et al., 2023; Li et al., 2025), other eye-movement measures may capture additional cognitively meaningful processes. Future studies could examine a broader set of eye-tracking metrics to further elucidate the role of EP in early reading development.

In conclusion, the present findings demonstrated that orthographic knowledge serves as a mediator in the association between children’s attention to words in environmental print and their reading ability. This result not only corroborates the pivotal role of environmental print in early reading development, but also advances the understanding how attention to words in EP is related to reading ability in young children.

## Data Availability

The data that support the findings of this study are available from the corresponding author upon reasonable request.
