# Supplementary material for: Orthographic knowledge mediates the link between environmental print attention and early reading ability
Source: Front Psychol. 2026 Jul 23;17:1852070. doi: 10.3389/fpsyg.2026.1852070 (PMC13442390; doi:10.3389/fpsyg.2026.1852070)
Supplement: Supplementary file 1 [file Supplementary_file_1.docx]

Supplementary Materials

To ensure the robustness of our findings and address potential confounding effects of item-level visual characteristics, we present supplementary item-level variance evaluations and participant-aggregated mediation analyses. We utilized the lme4 package (Bates et al., 2015) in R to construct unconditional null Linear Mixed-Effects Models (LMMs), in order to rigorously quantify the proportion of variance in children's eye-tracking metrics attributable exclusively to the physical heterogeneity of the stimulus items. The mediation models presented in this supplement were executed using the lavaan package in R. While the primary analyses in the main manuscript utilized the SPSS PROCESS macro (based on Ordinary Least Squares estimation), the lavaan implementation utilizes Maximum Likelihood (ML) estimation. This approach facilitates a fully reproducible, code-based workflow and yields completely standardized path coefficients to better illustrate relative effect sizes. The core statistical conclusions regarding the significance of the mediation pathways remain highly consistent across both analytical platforms.

**1. Baseline Item-Level Variance Evaluation via Linear Mixed-Effects Models (LMM)**

For each core eye-tracking metric, we fitted an intercept-only model specifying both participants (ID) and stimulus items (PID) as crossed random effects. The generic R formula applied was: Metric ~ 1 + (1 | ID) + (1 | PID). By extracting the variance components from these null models, we calculated the item-level Intraclass Correlation Coefficient (ICC). This metric represents the percentage of baseline systematic variance driven solely by item-level physical differences, independent of participant characteristics. The empirical proportions of baseline item variance (ICC_item_) for each metric are as follows:

- EP1 (Latency of first fixation): 13.72%
- LG10_EP2 (Percentage of fixation durations): 14.42%
- LG10_SP1 (Latency of first fixation in Control): 12.07%
- SP2 (Percentage of fixation durations in Control): 24.87%

In the Experimental Print (EP) conditions, item-level variance was maintained at a modest baseline (approximately 12-14%). This confirms that the authentic brand logos, despite their natural physical heterogeneity, did not introduce severe stimulus-driven attentional biases, maintaining an acceptable level of ecological noise. Conversely, item variance substantially increased in the Standard Print (SP) condition (approaching 25% for SP2). This validates our manipulation: removing contextual brand cues renders children's visual attention highly susceptible to bottom-up physical variations.

**2. Mediation analysis results with lavaan package**

The path diagrams and detailed parameter estimate for all mediation models (controlling for chronological age) are presented in Figure S1 and Table S1, respectively.

 **Figure S1.** Path diagrams of the direct and indirect effects of children's attention towards words on reading ability across Experimental Print (EP) and Standard Print (SP) conditions.

*Note. Completely standardized path coefficients are presented. Solid lines indicate significant paths (p < .05), whereas dotted lines indicate non-significant paths (p > .05). Chronological age was included as a covariate across all models. *p < .05, **p < .01, ***p < .001*

**Table S1.** Standardized and unstandardized estimates for the mediation models.

| Direct and indirect paths | Unstandardized β | Standardized β | 95% CI | |
| --- | --- | --- | --- | --- |
|  |  |  | Low | High |
| **the latency of the first fixation** |  |  |  |  |
| Path 1: EP 1 → Reading ability | -0.019** | -0.365 | -0.032 | -0.008 |
| Path 2: EP 1 → Orthographic knowledge → Reading ability | -0.010** | -0.186 | -0.016 | -0.003 |
| **the percentage of fixation durations** |  |  |  |  |
| Path 1: EP 2 → Reading ability | 24.752 | 0.190 | -4.208 | 54.986 |
| Path 2: EP 2 → Orthographic knowledge → Reading ability | 20.363* | 0.157 | 3.316 | 37.531 |
| **the latency of the first fixation** |  |  |  |  |
| Path 1: SP 1 → Reading ability | -5.429 | -0.038 | -36.038 | 27.857 |
| Path 2: SP 1 → Orthographic knowledge → Reading ability | -7.573 | -0.053 | -32.877 | 15.497 |
| **the percentage of fixation durations** |  |  |  |  |
| Path 1: SP 2 → Reading ability | 28.401* | 0.213 | 3.549 | 55.596 |
| Path 2: SP 2 → Orthographic knowledge → Reading ability | 17.598 | 0.132 | -1.319 | 35.148 |

*Note: Unstandardized β represents the raw path estimates, while Standardized β reflects the completely standardized effect sizes. 95% CIs are bias-corrected bootstrap confidence intervals for the unstandardized indirect effects based on 1000 resamples. Chronological age (in months) was included as a covariate on both the mediator and the dependent variable across all models. *p < .05, **p < .01, ***p < .001.*

*.*

The supplementary path models confirm that cognitive pathways in the EP condition remain highly robust, with the percentage of fixation durations (EP2) demonstrating a strict full mediation effect through orthographic knowledge. In contrast, the SP condition exhibits statistical vulnerability; the indirect pathways fail to reach significance in the decontextualized text conditions (95% CIs include zero). This structural divergence robustly corroborates the conclusion that authentic visual contexts are structurally indispensable for activating orthographic processing in young children.

**3. Original Experimental Stimuli**

To ensure complete transparency and address the reviewer's inquiries regarding visual complexity and ecological validity, the full set of original stimulus images used in both the Experimental Print (EP) and Standard Print (SP) conditions is provided below.

| Names in China |  | Description |
| --- | --- | --- |
| Oreo  (奥利奥) |  | A kind of biscuit of the Oreo brand |
| Cai Hong  (彩虹) |  | A kind of candy of the Skittles brand |
| Gong Gong Ce Suo  (公共厕所) |  | A public sign indicating a public toilet |
| Hai Di Xiao Zong Dui  (海底小纵队) |  | A cartoon named *The Octonauts* |
| Jin Zhi Xi Yan  (禁止吸烟) |  | A public sign of No Smoking |
| Ken De Ji  (肯德基) |  | A restaurant of fast food (KFC) |
| Wang Wang  (旺旺) |  | A snack of the Want Want brand |
| Wang Zai Niu Nai  (旺仔牛奶) |  | A kind of milk beverage of the Wangzai Milk brand |
| Xiao Zhu Pei Qi  (小猪佩奇) |  | A cartoon named *Peppa Pig* |
| Xiong Chu Mo  (熊出没) |  | A cartoon named *Boonie Bears* |

**Table S2** The EP with the top 10 familiarity

Note: To avoid copyright issues, the original trademark logos are not displayed. They are replaced with brief textual descriptions.

**References**

Bates, D., Mächler, M., Bolker, B., & Walker, S. (2015). Fitting Linear Mixed-Effects Models Using lme4. *Journal of Statistical Software*, 67(1), 1-48.

Rosseel, Y. (2012). lavaan: An R Package for Structural Equation Modeling. *Journal of Statistical Software*, 48(2), 1-36.
